# Deep sternal wound infection – latissimus dorsi flap is a reliable option for reconstruction of the thoracic wall

**DOI:** 10.1186/s12893-019-0631-4

**Published:** 2019-11-21

**Authors:** Nick Spindler, Stefanie Kade, Ulrich Spiegl, Martin Misfeld, Christoph Josten, Friedrich-Wilhelm Mohr, Michael Borger, Stefan Langer

**Affiliations:** 1 Department of Orthopedic Surgery, Traumatology and Plastic Surgery, University Hospital Leipzig, Liebigstrasse 20, 04103 Leipzig, Germany; 2 Department of Cardiac Surgery, Leipzig Heart Center, University of Leipzig, Leipzig, Germany

## Abstract

**Background:**

At present, data describing patients’ long-term outcomes, quality of life, and survival after deep sternal wound infection are rarely available.

The purpose of our study was to evaluate functional outcome and patient well-being after debridement and reconstruction of the sternal defect using a pedicled latissimus dorsi flap following deep sternal wound infection (DSWI).

**Methods:**

This retrospective analysis reviewed 106 cases of DSWI after open-heart surgery treated between May 1, 2012, and May 31, 2015. The parameters of interest were demographic and medical data, including comorbidity and mortality. Follow-up consisted of physical examination of the patients using a specific shoulder assessment, including strength tests and measurements of pulmonary function.

**Results:**

The population consisted of 69 (65%) male and 37 (35%) female patients. Their average age at the time of plastic surgery was 69 years (range: 35–85). The 30-day mortality was 20% (*n* = 21); after one-year, mortality was 47% (*n* = 50), and at follow-up, it was 54% (*n* = 58).

Heart surgery was elective in 45 cases (42%), urgent in 31 cases (29%) and for emergency reasons in 30 cases (28%). The preoperative European System for Cardiac Operative Risk Evaluation (EuroSCORE) averaged 16.3 (range: 0.88–76.76).

On the dynamometer assessment, a value of 181 Newton (N) (±97) could be achieved on the donor side, in contrast to 205 N (±91) on the contralateral side.

The inspiratory vital capacity of the lung was reduced to an average of 70.58% (range: 26–118), and the forced expiratory volume in 1 s was decreased to an average of 69.85% (range: 38.2–118).

**Conclusions:**

Given that only small adverse effects in shoulder function, strength, and pulmonary function were observed, the latissimus dorsi flap appears to be a safe and reliable option for the reconstruction of the sternal region after DSWI.

## Background

Although Milton first described median sternotomy at the end of the nineteenth century as a reliable approach to the ventral mediastinum, and it became standardized for use in cardiac interventions in 1956, the treatment of deep sternal wound infections (DSWI) and reconstruction of the mediastinal region have remained a challenge for plastic surgeons [1]. Postoperative mediastinitis after cardiac surgery is still a random but devastating complication. The incidence ranges between 0.5 and 4%, with a mortality rate of up to 50% [2–6]. Extensive morbidity and the need for multiple revisions, the weak perfusion of the remaining sternum, and the long-term antibiotic treatment and hospitalization associated with this population result in high costs to the health care system [7]. Common risk factors are male sex, age over 73 years, the use of one or both internal mammary arteries as by-pass grafts, obesity, diabetes mellitus, chronic obstructive pulmonary disease, blood transfusion and the length of the primary cardiac operation [8, 9]. As these risk factors accumulate, the prognosis deteriorates considerably, resulting in severe osteomyelitis of the sternum, an unstable thorax or destructive DSWI [10]. Although different flaps are available to the plastic surgeon for wound closure, there is no standard operating procedure for patients with DSWI. However, there is broad consent that the basic principle of treatment consists of radical sternal wound debridement and coverage with well-vascularized tissue, which serves as a carrier to transport antibiotics to their target location the mediastinal region [11–15]. The most common concept of treatment consists of a multistage procedure. The reconstruction of the sternal defect takes place after multiple debridements and conditioning of the wound using negative pressure wound therapy (NPWT) has been performed [16, 17]. In contrast, we debride and reconstruct the majority of the sternal wounds simultaneously, using the latissimus dorsi muscle as a pedicled myocutaneous flap.

At present, data describing patient satisfaction, long-term quality of life after surgery, and overall survival of this specific population are rarely available.

The purpose of our study was to evaluate functional outcomes and patient well-being after the debridement and simultaneous reconstruction of the sternal defect using a pedicled latissimus dorsi flap.

## Methods

The study was approved by the Ethical Committee at the Medical Faculty, Leipzig University, Leipzig, Germany.

This retrospective analysis reviewed 106 cases of DSWI after open-heart surgery treated during a three-year period from May 1, 2012, to May 31, 2015**,** at the Department of Orthopaedic Surgery, Traumatology and Plastic Surgery at the University Hospital Leipzig in Leipzig, Germany.

Follow-up was performed 18 months after discharge from the hospital. The parameters of interest were demographic and medical data, including comorbidity and preoperative evaluation using the European System for Cardiac Operative Risk Evaluation (EuroSCORE). Mortality, postoperative complications as cardiac and pulmonary failure due to cardiac surgery as well as wound healing disorders due to plastic reconstructive surgery were recorded. The results of the general physical examinations, shoulder assessment using the Constant and Murley score, the Short Form Health Survey (SF-36) questionnaire, dynamometer assessment, and the results of the body plethysmography were also taken into account and evaluated.

The inclusion criteria were as follows: patients suffering from DSWI after median sternotomy due to cardiac surgery, including coronary by-pass surgery, valve replacement or reconstruction alone or in combination with by-pass grafting, aortic bow replacement, heart or heart-lung transplantation, and age greater than or equal 18 years. All included patients had to fulfill the Centers for Disease Control and Prevention (CDC) criteria for DSWI showing either purulent discharge from the wound, fever (> 37,5 °C), leucocytosis greater than 10.000/μl or an open thorax [18].

The initial examination of the infected wound after the diagnosis of DSWI was generally performed by the cardiac surgeon. The wound was conditioned using NPWT, and the patient was transferred to our unit for further treatment. The subsequent therapy consisted of radical local debridement of the infected and necrotic soft tissue and the bone material. Debridement was performed until a well-vascularized wound ground and bleeding bone edges were exposed. Therefore, partial or even total resection of the sternum had to be performed. Artificial material, such as Robicsek cerclages, was always removed, and the mediastinum was debrided. Reconstruction of the thorax was performed simultaneously using a pedicled, myocutaneous latissimus dorsi flap.

**Fig. 1:**
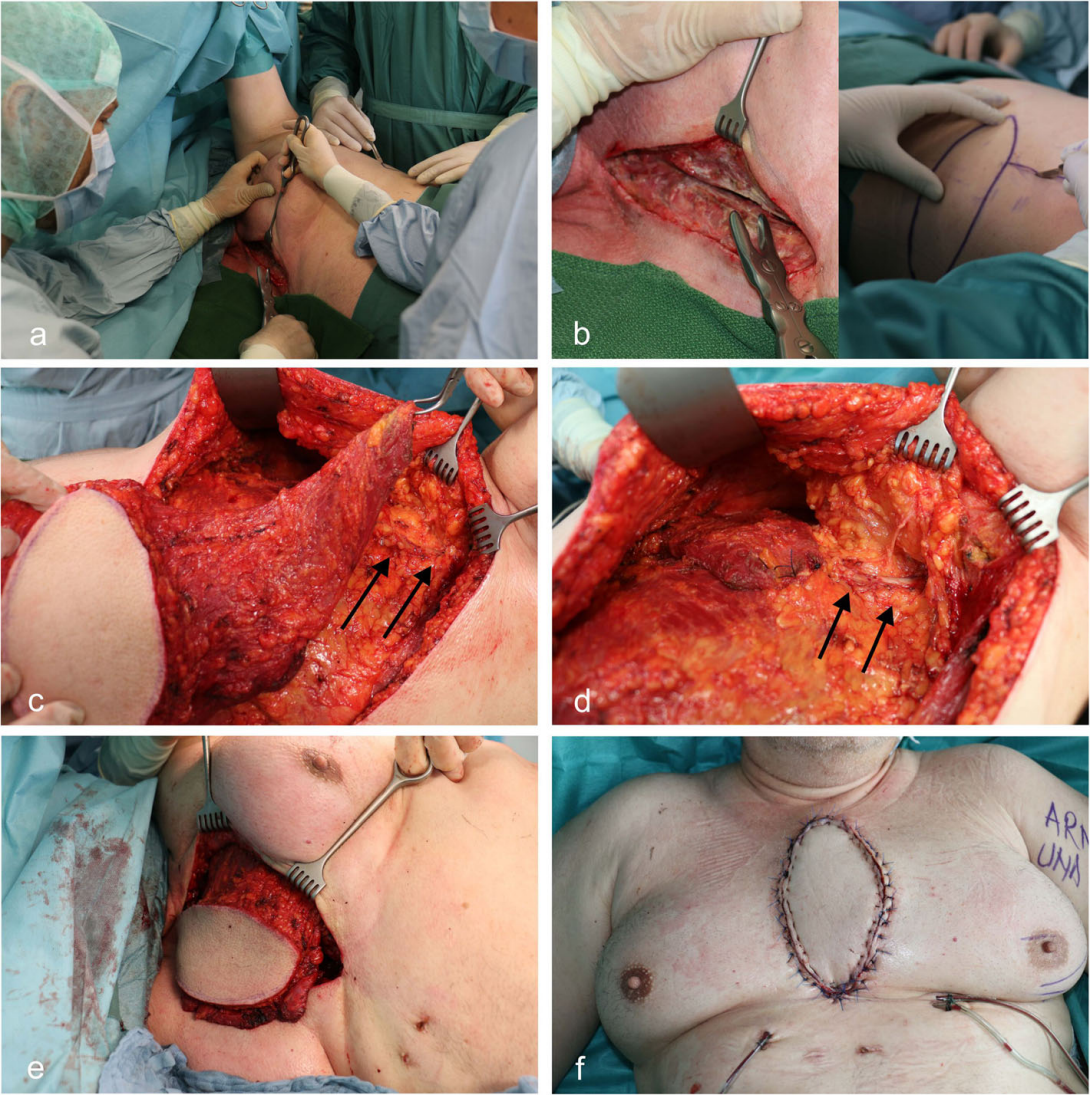
**a** Side positioning of the patient making a two team approach possible **b** Debridement of the sternum and harvesting the latissimus dorsi flap with a 18x7cm skin island simultaneously **c** Fully harvested myocutaneous flap solely connected to the body by a skeletonized 12 cm long pedicle (double arrow) making a wide advancement of the flap into the anterior mediastinal region possible **d** Fixation of the tendinous part of the latissimus muscle on the thorax wall preventing the vessels from uncontrolled tension and movement (double arrow) **e** The muscular part of the flaps fills up the deep cavity of the mediastinum and the skin island makes a relaxed closing of the already retracted skin edges possible **f** 1 Week post operatively the flap shows an excellent state of perfusion without any signs of wound healing disorder

The patient was placed in a side position, and the arm was abducted at a 90° angle (Fig. 1a + b). In this position, the mediastinum can be debrided, and the flap can be harvested simultaneously by a second surgeon. The vessels were skeletonized until their derivement from the subclavian artery and vein to create a long vascular pedicle. A one centimetre-measuring piece was cut out of the accompanying nerve to create a denervated flap. Hereafter the origin of the muscle was separated from the humerus and the muscle flap could be placed easily in the anterior mediastinum (Fig. 1c). After the latissimus dorsi muscle was transferred through a subcutaneous tunnel to the ventral mediastinum, the tendinous part was stapled to the thorax wall to protect the pedicle from shear forces. The vessels were laid on a fatty bed without tension or compression (Fig. 1d). Over two suction drainages, the muscle was sutured into the anterior mediastinum (Fig. 1e). After the positioning of subcutaneous suction drainages, wound closure was approached. No osteosynthetic stabilization of the remaining parts of the sternum or ribs was performed in any patient. Stability of the thorax developed with the scarification of the latissimus dorsi muscle over time (Fig. 1f).

### Physical examination

General physical examinations (*n* = 39) evaluated aesthetic results, pain and sensitivity. To examine the function of the shoulder, an assessment using the Constant and Murley score was performed. To assess patient health status, the Short-Form Health Survey (SF-36) questionnaire was used. Interviews were conducted during regular clinical follow-up [19].

### Dynamometer assessment

Dynamic and isometric measurements (*n* = 31) with a modified latissimus pull exercise were used to measure the strength of the shoulder. The measurements were repeated three times on each side, and the mean values were calculated and compared to the contralateral, non-donor, side.

### Pulmonary function

Measurements of pulmonary function (*n* = 37) using volume displacement body plethysmography were carried out by comparing the results to physiological reference values.

## Results

From May 2012 until May 2015, we treated 106 patients suffering from DSWI. The population consisted of 69 (65%) male and 37 (35%) female patients. The average age at the time of plastic surgery was 69 years (range: 35–85 years). In our population, 94% of the patients had arterial hypertonia, 59% had diabetes mellitus and 46% were obese. Heart surgery took place electively in 45 cases (42%), urgently in 31 cases (29%), and for emergency reasons in 30 cases (28%). The preoperative EuroSCORE was 16.3 (range: 0.88–76.76).

By-pass surgery alone (1–5 vessels) was performed in 32 patients (60%) off pump and in 21 patients (40%) receiving extra-corporal circulation (ECC). Nine patients (8%) received a single-valve replacement and reconstruction; in 10 cases (9%) the operation was performed on two valves. Nineteen patients (18%) received by-pass surgery in combination with a valve replacement or reconstruction. In 8 patients (8%), an aortic bow replacement with replacement of the aortic valve was performed; three patients (3%) received a heart, one (1%) received a heart-lung transplantation, one (1%) received an external heart assistive device, and two patients (2%) underwent open surgery of the pulmonary arteries due to embolism.

Our population represents the characteristic risk profile for DSWI (Table 1).

**Table 1:** Risk Profile of the patients developing a DSWI

Patients risk profile	Incidence
	*n* = 106 (%)
Age (y)	69
Male	69 (65,0)
Female	37 (35,0)
Arterial hypertension	94 (88,6)
New York Heart Assoziation (NYHA)	
NYHA I	14 (13,2)
NYHA II	26 (24,5)
NYHA III	52 (49,1)
NYHA IV	14 (13,2)
Type 2 diabetes mellitus (T2DM)	59 (55,6)
Obesity	49 (46,2)
I [BMI 30,0–34,9 kg/ m^2^]	28 (26,4)
II [BMI 35,0–39,9 kg/ m^2^]	15 (14,1)
III [BMI ≥ 40,0 kg/ m^2^]	6 (5,6)
Hyperlipoproteinaemia	72 (67,9)
Chronic obstructive pulmonary disease	7 (6,6)
Status post myocardial infarction	42 (39,6)
Nicotine abuse	42 (39,6)

After the initial cardiac surgery, nine patients (8%) needed cardiac reanimation, arrhythmia was observed in 45 patients (42%), and in 31 cases (29%), patients needed intermediate haemodialysis. Postoperatively, ventilation failure occurred in 59 cases (56%), and 24 patients (23%) needed a tracheotomy and extended ventilation. The overall ventilation time averaged 282 h (range: 4.88–3628.22).

The defects were all reconstructed using a pedicled latissimus dorsi flap. We had no total flap loss in any patient. Thirty-five percent needed sternal revision due to wound healing disorder, haematoma or persistent infection. Twenty percent of our patients showed post-operative bleeding.

The 30-day mortality from the time of the sternal reconstruction was 20% (*n* = 21), the one-year mortality was 47% (*n* = 50), and at the time of the study, 18 months after discharge from the hospital, 54% (*n* = 58) had died. Nine (8%) patients could not participate in the study due to a reduced state of health. Thirty-nine (37%) patients were clinically examined and questioned using the SF-36 questionnaire. Due to reduced physical state, only 31 patients could perform the dynamometric test reliably. Two patients were examined ex domo, and therefore, the standardized pulmonary function test could not be completed.

### Physical examination

In the physical examination (*n* = 39), no clinical signs of local infection or residuals of any infection could be recognized. The skin island had an average size of 18.6 × 5.8 cm.

No patients showed any signs of thorax compression pain. Four patients experienced hypoesthesia in the sternal region, 4 patients experienced painful scarring, and 12 patients reported pain upon rotation of the upper body. Fifteen patients suffered from tightness of the chest, and seven reported intermediate crepitation. Fifteen patients feared a possible fall or being hit on the chest.

**Fig. 2:**
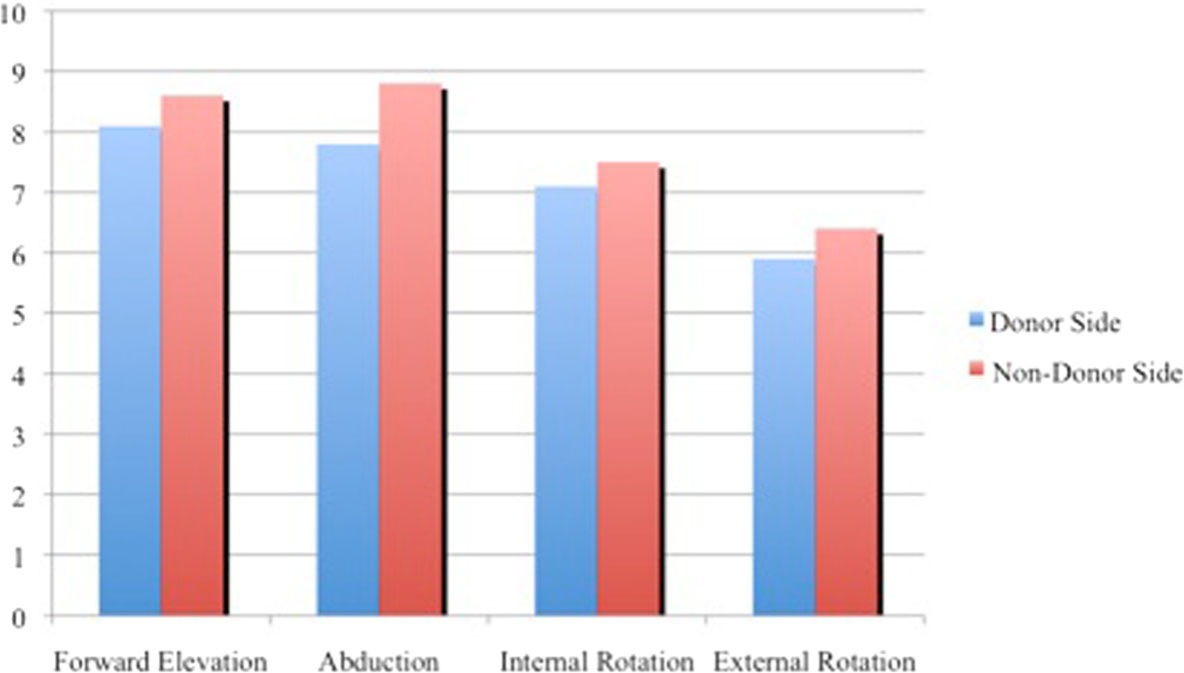
Constant and Murley score: Range of motion (min. 0 – max. 10)

According to the Constant and Murley score, the range of motion showed no significant difference between the donor side and the contralateral side (Fig. 2).

### Quality of life index

**Fig. 3:**
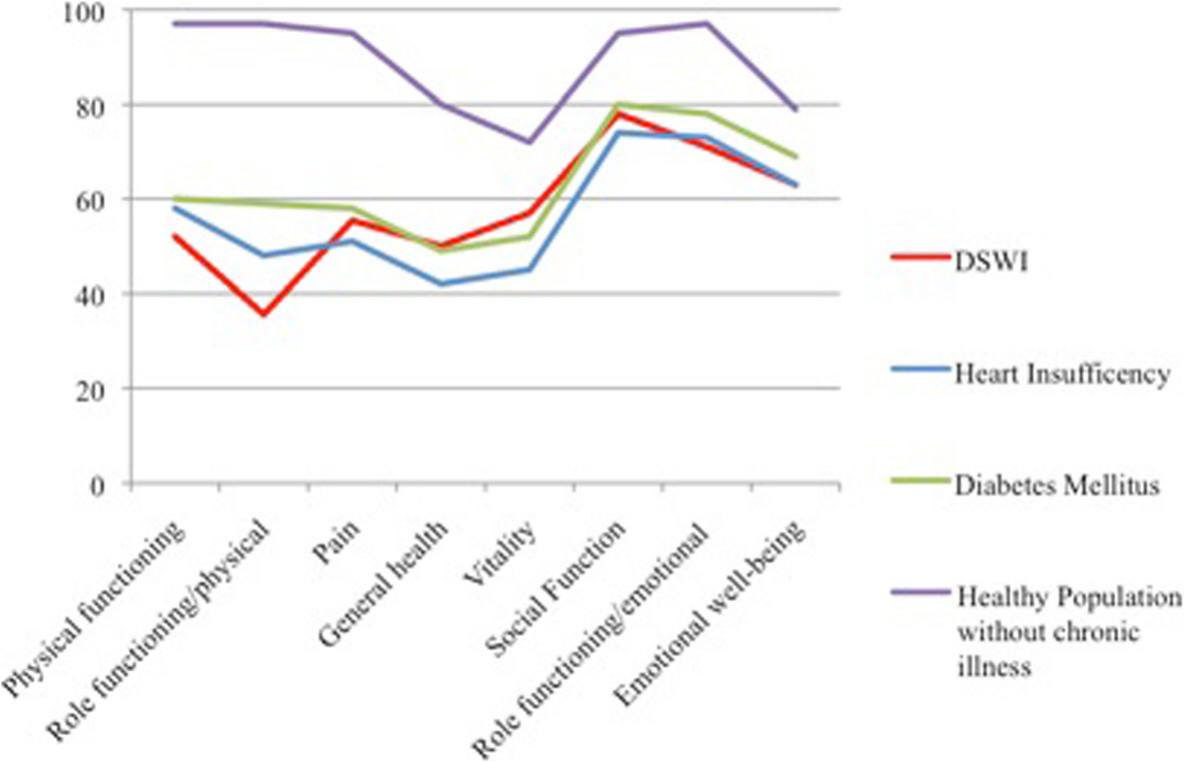
Results of the Quality of life questionnaire on DSWI Patients in contrast to other chronic diseases and a healthy population. (Values for heart insufficiency, diabetes mellitus and the healthy compare population have been taken from Bullinger et al. [19])

**Fig. 4:**
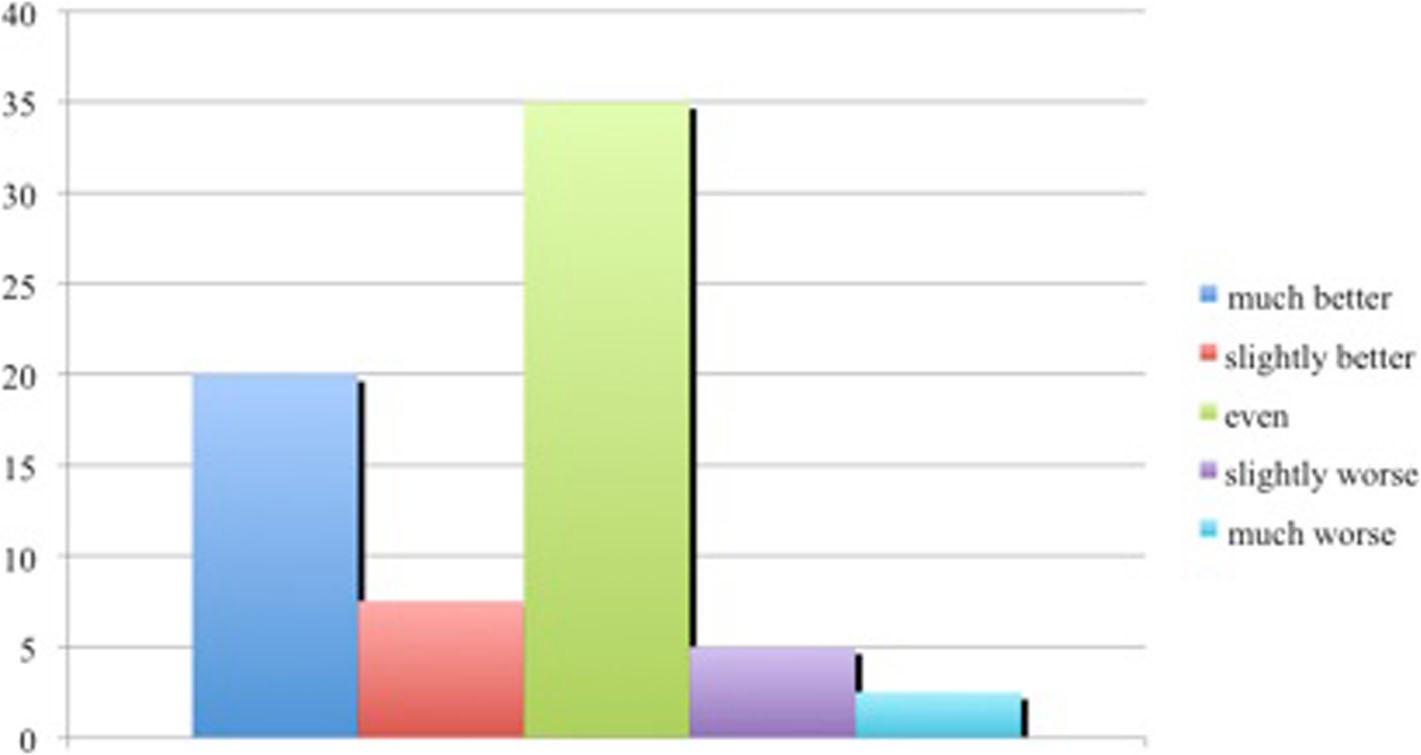
Self-evaluated classifications of the patients acute state in comparison to prior the plastic reconstruction (declaration in percent)

The quality of life assessment (n = 39) showed a decrease in nearly all categories. Compared with the findings for chronic obstructive pulmonary disease (COPD) or heart insufficiency, the curves demonstrate a clear decrease due to the patients’ general health status, especially in the categories of physical functioning and role limitation. Fig. 3 shows a summary of the findings and illustrates the course in comparison to reference values for diabetes mellitus and heart insufficiency and representative values for a healthy population without chronic illness investigated by Bullinger et al. [20] The patients also completed a self-assessed evaluation of their personal state of well-being (Fig. 4).

### Dynamometer test

**Fig. 5:**
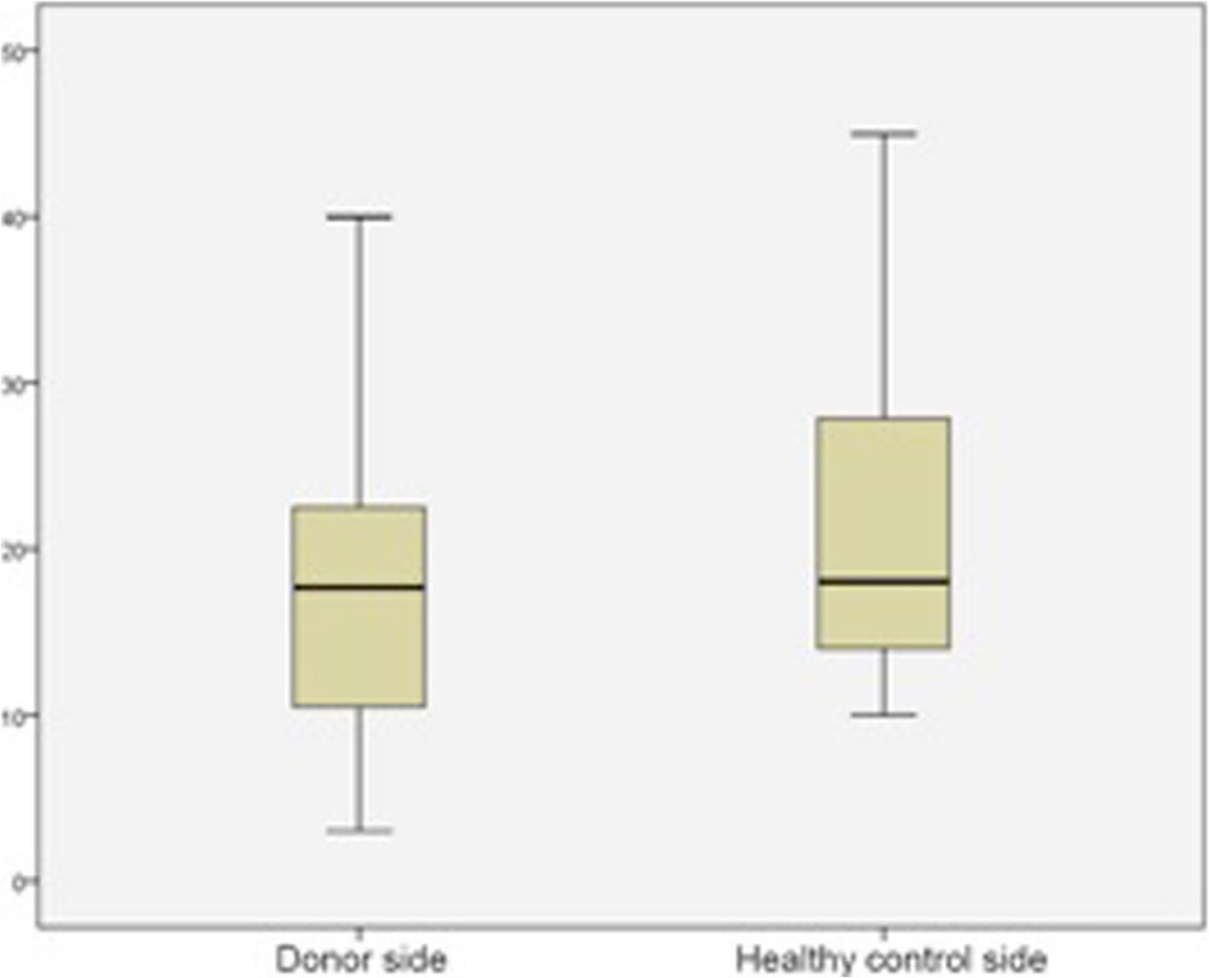
Dynamometer investigation of the latissimus dorsi pulling test comparing the donor side - with the healthy contra lateral side

In the dynamometer examination (*n* = 31), the patients reached a mean strength of 197 Newton (N) (±86) on the right side and 182 N (±97) on the left side. Additionally, there were two aberrations recorded that reached 400 N and 450 N. Comparing the donor side to the contralateral side, where no muscle had been harvested, the donor side achieved a mean pull of 181 N (±97), in contrast to 205 N (±91) on the non-operated side (Fig. 5).

### Pulmonary function

Thirty-six patients performed the pulmonary function test. The inspiratory vital capacity of the lung showed a reduction to an average of 70,5 (range: 26–118), the forced vital capacity showed similar results averaging 70,4 (range: 27–118), and the forced expiratory volume in 1 s was decreased to an average of 69,8 (range: 38,2–118). The Tiffeneau Test, on the other hand, showed an average of 99,9 (range: 63–139). Twenty-six patients showed a restrictive pulmonary ventilation disorder, with only 4 having an obstructive disorder. The vital capacity of the lung showed an average of 77% in the subpopulation with a partially resected sternum in comparison to 64% in the group with a complete sternal resection. Further the forced vital capacity showed an average of 79% in the group with partial debridement to 64% in the other group. The mean expiratory flow of 75% was similar in both groups, 58 and 62% respectively.

## Discussion

Deep sternal wound infections (DSWI) are still devastating complications after median sternotomy following cardiac surgery. Despite a low incidence, the high revision rates and extended hospitalization of the patients increase the economic impact to the health care system [2–7, 21, 22]. Fu et al. showed that the resulting cost exceeds that of the initial cardiac surgery by a factor of three [23]. The presence of the known risk factors arterial hypertonia, diabetes mellitus and obesity substantially increases the incidence of DSWI [24, 25]. Our population was affected by all the risk factors that predict a higher occurrence of DSWI [8, 26–28].

The two main targets in DSWI treatment are control of the infection and reconstruction of the thorax. Therefore, the early radical debridement of the sternum and mediastinum is essential to the outcome and reduces the mortality of patients suffering from mediastinitis [29]. Total resection of the sternum has only been performed in cases of massive bone necrosis in our population. However, after debridement of the sternum, the edges sometimes pressed into the right ventricle. To avoid the possible perforation of the heart, extended resection of the sternum was required in some cases.

The former unsatisfying therapy concept of open granulation or closed lavage using suction systems was revolutionized 1969 by the idea of reconstructing the sternal defect using pedicled muscle flaps [30]. Currently, the plastic surgeon has access to a large variety of different flap options for reconstructing the anterior mediastinum [11]. Furthermore, NPWT has become a modern option in wound treatment and is especially popular for temporary sealing [31]. It prevents cutaneous retraction and supports the patients’ ability to breathe spontaneously by giving the thorax more stability. The foam used can be directly placed on the heart and works as a cushion to protect the ventricle from perforation by the sharp edges of the longitudinally cut sternum [32, 33]. However, NPWT is only a temporary solution until definite reconstruction of the chest wall is established.

The ideal technique for reconstruction of the sternal area has been the subject of controversy for many years [34–37]. The use of a myocutaneous latissimus dorsi flap allows us to reconstruct the potentially superinfected mediastinal area with unaffected tissues. Moreover, especially during prolonged intensive care treatment, atrophy of the muscles immediately sets in. The thin pectoralis muscle is mainly affected, especially after prolonged ventilation, as we observed in 56% of our population. Daigeler et al. found that 1 year after reconstruction using a pectoralis flap, the muscle had lost 1/3 of its original volume, and coverage, especially in the lower third of the sternum, was only rarely present [38]. Therefore, we prefer the latissimus dorsi flap for the reconstruction of sternal defects.

Radical debridement of the bone and soft tissue is performed simultaneously with the reconstruction of the thorax. The dead space in the ventral mediastinum is filled up using a pedicled myocutaneous latissimus dorsi flap [17, 39, 40]. Currently, the operative approach follows the surgeon’s preference as there is no robust clinical evidence favouring a simultaneous vs. a multistage treatment [41, 42]. The rate of complications due to wound dehiscence or post-operative bleeding is caused by persistent infection, therapeutic anticoagulation and the patients’ comorbidity. However, revision rates are comparable to those of other large series using multistage treatment [38]. The limited data concerning the time period between cardiac and reconstructive surgery as well as the stage of osteomyelitis makes it difficult to compare different series.

The 30-day mortality after plastic surgery was 20% in our population and is comparable with other, larger series [35, 43]. The one-year mortality rate of 47% and the mortality rate of 54% at follow-up are distinctly high. However, our population had a worse state of health than those of the referred series. A pronounced degree of comorbidity and the combined existence of all relevant risk factors already indicated a poor prognosis overall. Furthermore, our population also showed a high EuroSCORE (average: 16), indicating the presence of life-threatening situations. The operative procedure and the intraoperative change of bearing were generally tolerated without any problems. Certainly, the high rate of seroma and postoperative bleeding led to an impairment of the general condition. However, the patients showed no signs of DSWI according the CDC criteria during follow-up. Therefore, due to the long period between surgery and death, the reconstruction of the chest wall is not considered etiological. Moreover, the accompanying illness of the patients and decompensation of heart and pulmonary function caused this high mortality rate.

Reconstruction of the thorax wall after DSWI is still a challenge characterized by the fundamentals of plastic surgery: the need to restore form and function with minimal donor side effects.

Shoulder function is the result of the complex interaction of 27 different muscles. The latissimus muscle is one of the body’s largest muscles. It has 6 synergists that assist the shoulder with medial rotation, abduction, extension, depression of the arm, downward rotation of the scapula and further stabilization of the humeral head as a secondary function [44–49]. Using the DASH score, two studies assessed the impairment of the shoulder after plastic reconstruction using the pedicled latissimus dorsi flap. The results revealed that the duration of impairment differs, with substantial morbidity for the patient [50, 51]. In contrast, Hankins et al. showed in their seven-year follow-up that there were no subjective complaints of shoulder impairment, mobility or weakness [52]. However, physical therapy plays a significant role in post-operative care of patients undergoing reconstruction with a latissimus dorsi flap [49].

This reflects the findings for our population. An examination using the Constant and Murley Score in our population showed no major alteration in the function of the shoulder on the donor side compared to the healthy non-donor side. In the dynamometer testing, the strength parameters of the donor side did not differ significantly from those of the contralateral side. One reason for this finding is that the synergists compensate for the function and strength of the latissimus dorsi muscle. However, handedness had an additional impact. In 82% of the cases, the latissimus dorsi flap was harvested from the left side. The predominant side in Germany is the right hand in over 90% of the population; therefore, this side is already stronger by nature [53]. Therefore, the results showed only a physiological difference between the donor and the healthy contralateral non-donor side.

Our population reported an undefined feeling of tightness of the thorax, which we ascribed to scarification of the muscle and the recovery of thoracic compliance. There were no signs of thorax instability or paradox breathing in the follow-up and only 3 patients reported slight compression pain of the thorax. However, the pure fear of possible pain was a concern of the patients. In contrast to other series, acute pain when changing the position of the upper body was reported by a rather large number of patients [54, 55]. The long period of hospitalization, the multimorbid state and the severe cardiac disease our population endured are explanations for the physical limitations in our group. We also attribute the fairly poor performance on the quality of life questionnaire to their long-lasting trauma. In particular, body function and control, which represent the patients’ physical capability in daily life, were rated poorly. These findings were similar to those of Daigeler et al., who evaluated 24 patients after DSWI and reconstructed the mediastinum using pectoralis major flaps [38]. Therefore, the use of the pectoralis or the latissimus dorsi flap does not seem to have a large influence on the patient’s quality of life. Moreover, the long-term process of disease is responsible for the reduced state of health in patients. Both muscles work as auxiliary respiratory muscles, which results in the described reduced values of the pulmonary function test. Moreover, long-term exposure to multiple surgical interventions, prolonged hospitalization and slow rehabilitation without achieving a restitutio ad integrum are reasons for the impaired quality of life of our population.

Nonetheless, our participants were in a very good psychological state. In comparison to other large series, more than half of our patients rated their actual state as good as or better than before the operation, and only 18% were not satisfied with the result achieved or reported a lack of sensitivity [56, 57].

In contrast to other series, where either dynamic or static parameters of the pulmonary function test were impaired, our population showed a distinctive decrease in both categories. The forced expiratory vital capacity and the forced expiratory volume in 1 s were approximately 10% below the physiologic border and were similar to the findings of other groups [12, 38, 58, 59]. Additionally, the static values were slightly decreased, indicating that the biomechanical stability of the thorax was restored; however, full functional restitutio could not be achieved due to the patients’ multimorbid state. Cohen et al. performed pulmonary function analysis pre- and post-operatively and showed that after reconstruction, the pulmonary function improved considerably [60].

In our opinion, it is therefore mandatory to fill the mediastinal gap with a voluminous muscle and give the mediastinal area the opportunity to build a firm scar and thereby restore the compliance of the thorax. Because the inferior third of the sternal region can hardly be covered with the pectoralis flap, we regularly use the latissimus dorsi flap [61, 62]. It supports the reconstructive surgeon with a wide and long skin island and enough residual muscle to fill the anterior mediastinum of all three parts of the sternum [36, 63].

### Limitations

Nonetheless, this study has some limitations. Due to the high mortality rate, we could only evaluate a reduced part of the original population. This limited number of patients in our series precludes sophisticated statistical analysis. Furthermore is the retrospective design and the short follow-up time a limiting factor.

## Conclusion

Functional impairment after debridement of the sternum and reconstruction using a pedicled latissimus dorsi flap is considerable. However, the sequelae are not to be attributed to the reconstruction alone but are caused by the multimorbid state and the underlying illness of the patient.

The fact that we observed few adverse effects in shoulder function, strength and pulmonary function, as well as the long vascular pedicle of this wide and voluminous muscle, makes the latissimus dorsi flap a reliable option for reconstruction of the thorax after DSWI.

## Data Availability

The datasets used and analyzed during the current study are available from the corresponding author on reasonable request.

## Abbreviations

CDC: Centers for Disease Control and Prevention
COPD: Chronic Obstructive Pulmonary Disease
DSWI: Deep Sternal Wound Infections
ECC: Extra-Corporal Circulation
EuroSCORE: European System for Cardiac Operative Risk Evaluation
N: Newton
NPWT: Negative Pressure Wound Therapy
